# Gluteus maximus tendon transfer as a salvage option for painful chronic hip abductor insufficiency: clinical and MRI results with a minimum follow-up of 24 months

**DOI:** 10.1177/11207000231197760

**Published:** 2023-09-05

**Authors:** Dominik Kaiser, Armando Hoch, Reto Sutter, Patrick O Zingg

**Affiliations:** 1 Department of Orthopaedics, Balgrist University Hospital, Zurich, Switzerland; 2 Department of Radiology, Balgrist University Hospital, Zurich, Switzerland

**Keywords:** Hip abductor insufficiency, hip abductor reconstruction, hip abductors, total hip arthroplasty, Trendelenburg gait, Whiteside transfer

## Abstract

**Introduction:**

Chronic hip abductor insufficiency is a rare debilitating condition. In cases refractory to conservative treatment and not amenable to direct repair an augmentation becomes necessary. The preferred salvage method at our institution is augmentation with the anterior third of the gluteus maximus tendon. The aim of this study is to describe the results of 8 patients, treated for painful chronic hip abductor insufficiency with gluteus maximus muscle transfer, after a minimal follow-up of 24 months including a full clinical and MRI evaluation of the hip abductors pre- and postoperatively.

**Methods:**

We retrospectively reviewed a consecutive series of 8 patients who were surgically managed for painful chronic hip abductor insufficiency. All patients had a Trendelenburg sign, impaired muscle strength (M ⩽ 3) as well as a complete avulsion of the hip abductors with marked fatty degeneration (⩾3). Pain levels, muscle strength, functional scores as well as a postoperative MRI was obtained after a minimal follow-up of 24 months.

**Results:**

The mean age of the patients was 69 years, mean follow-up was 35 (26–54) months. Pain was significantly reduced postoperatively to VAS 2.5 from VAS 5 (*p* *=* 0.046). Trendelenburg sign remained positive in all patients and hip abductor strength did not improve significantly from 2.4 to 3.1 (*p* *=* 0.19). Complete healing of the transferred tendon was confirmed by MRI in all patients at last follow-up.

**Conclusions:**

In the setting of painful chronic hip abductor insufficiency refractory to conservative treatment with advanced muscle degeneration without the possibility of a direct reconstruction the gluteus maximus tendon transfer significantly decreased pain. The effect on hip abductor strength and patient-reported functional outcome scores is limited. Despite the modest results it remains our preferred salvage treatment option for lack of better alternatives. Larger studies are necessary to confirm these findings.

## Introduction

Greater trochanter pain syndrome (GTPS) is estimated to affect between 10% and 25% of the population in industrialised societies and is associated with inflammation and tears of either the gluteus medius and/or gluteus minimus in up to 20% especially in patients over the age of 50.^1–8^ These 2 muscles are the most important hip abductors and are crucial for maintaining pelvic stability and a normal gait.^9^

3 different clinical tear scenarios have been described including hip abductor tears found at the time of total hip arthroplasty (THA), hip abductor avulsion after THA *via* anterolateral or trans-gluteal approach as well as chronic, degenerative tears.^10,11^

After failed conservative treatment many open and endoscopic techniques have shown successful outcomes as long as the muscle degeneration is limited and the tendon can be adequately mobilised and repaired directly.^12,13^

**Figure 1.**
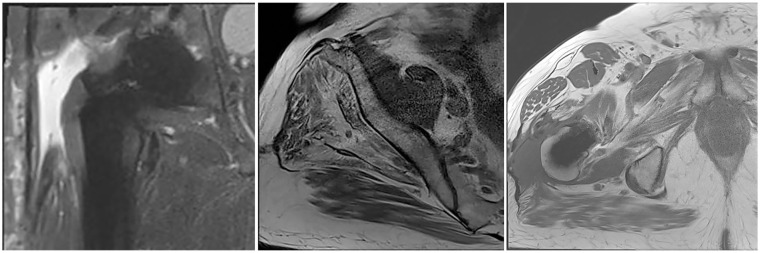
MR images depicting a complete avulsion of the gluteus medius and minimus tendon as well as a fatty degeneration of both muscles.

However, in the rare setting of a chronic abductor insufficiency not amenable to direct repair an augmentation becomes necessary (Figure 1).^14^ Several different techniques have been described with transfer of local muscles and or synthetic allografts.^13–19^ At our institution we prefer the augmentation of the anterior third of the gluteus maximus (GM) as the donor site morbidity is of little clinical relevance.^18^

Some clinical results for chronic hip abductor insufficiency are promising with improvement of hip abductor strength and significant improvement to pain level and clinical outcome scores after 12 months while others remain modest at best.^20–22^

However, the following questions remain unanswered:

(1) Will the clinical result remain satisfactory after a minimal follow-up of 24 months?
(2) Does the extraanatomical tendon transfer heal?
(3) Can it stop the fatty degeneration of the native hip abductor muscles?
(4) What happens to the transferred and the retained gluteus maximus muscle?

The aim of this study is to describe the clinical and functional results of 8 patients, treated for painful chronic hip abductor insufficiency with gluteus maximus muscle transfer, after a minimal follow-up of 24 months including a full clinical and magnetic resonance imaging (MRI) evaluation of the hip abductors pre- and postoperatively.

## Materials and methods

This study was approved by our ethical review board and all participants gave written informed consent (KEK-ZH: BASEC Nr.2020-00710).

### Study design and population

This retrospective, consecutive case series was conducted entirely at the author’s institution. A case recording form was established on which clinical and imaging data and therapeutic results were set down. A single author (AH) examined all patients as part of a clinical and radiological examination after a minimum follow-up of 24 months.

### Inclusion criteria

The patients were selected from January 2016 to June 2018. Inclusion criteria were adult patients with painful chronic hip abductor insufficiency refractory to our conservative management protocol including a minimum of 6 months of targeted physiotherapy, refractory to non-steroidal anti-inflammatory drugs (NSAIDs) and ⩾1 infiltration of the trochanteric bursa with triamcinolone 40 mg and lidocaine (2%) 100 mg, a hip abductor strength of ⩽M3 and an MRI showing marked fatty degeneration of the gluteus medius muscle (Goutallier ⩾ 3) as well a complete avulsion of the gluteus medius and minimus tendon (Figure 1).

8 patients were identified in whom a direct reconstruction was not considered feasible. Preoperatively all of these 8 patients had a positive Trendelenburg sign, Trendelenburg gait and a normal gluteus maximus (no atrophy of the gluteus maximus and a fatty infiltration ⩽2). The muscle atrophy and fatty degeneration was evaluated according to the revised Goutallier classification on MRI.^23–25^ A minimum follow-up of 24 months was required.

### Surgical intervention and rehab protocol

Surgery was performed in the lateral decubitus position. After longitudinal incision of the fascia lata, the trochanteric bursa was systematically removed to localise the tendon insertion. After debridement of the tendon and the greater trochanter, the tendon of the gluteus medius muscle and gluteus minimus muscle were identified. After intraoperative confirmation of the preoperative plan a gluteus maximus transfer was performed according to Whiteside.^14,18^ Postoperatively the patients were educated to walk with partial weight-bearing of 15 kg using 2 crutches. Reconstruction of the hip abductors was protected by a pelvis leg cast for 6 weeks. Hip flexion was limited to <70°, no active hip abduction or passive adduction was allowed for the first 3 months. Physiotherapy was initiated immediately. After 6 weeks progressive weight-bearing was initiated. All patients received standard anticoagulation therapy for 6 weeks.

### Patient evaluation

Preoperative evaluation was obtained from the patient records. The preoperative MRI was reevaluated by a specialised board-certified musculoskeletal radiologist (RS). The 3 gluteal muscles were analysed individually. Each muscle was divided into an anterior third, middle third and posterior third. Muscular atrophy was assessed on a scale from 0 to 3 (0: normal, 1: mild atrophy, 2: moderate atrophy, 3: severe atrophy) and fatty degeneration according to the modified Goutallier classification.^25^ All patients were evaluated at 6 weeks, 12 weeks, 1 year postoperatively and at latest follow-up after a mean of 35 months (26–54 months).

At the final evaluation visual analogue pain score (VAS),^26^ Harris Hip Score (HHS) scores,^27^ muscle strength according to the Medical Research Council (MRC) score and the presence of a Trendelenburg sign as well as gait abnormalities were evaluated by the same author (AH).^28^ An anteroposterior (AP) pelvic radiograph, a cross table axial view as well as an MRI were performed to evaluate the integrity/continuity of the repair and evaluate the difference in atrophy and fatty degeneration of the gluteal muscles compared to preoperatively.

### MRI

Pre- and postoperative MR images were obtained with a 3T Siemens Prisma MR scanner (Siemens Healthcare, Erlangen, Germany) with a combination of a spine coil and a body surface coil placed over the hip of the patient for unilateral imaging of the hip. The following MR sequences were acquired: coronal T2-weighted turbo-spin echo sequence (repetition time[TR]/echo time[TE] 4000 ms/83 ms; Field-of-view[FOV] 220 mm; matrix 448 × 448; slice thickness 4 mm), sagittal T1-weighted turbo-spin echo sequence (TR/TE 678 ms/14 ms; FOV 180 mm; matrix 512 × 512; slice thickness 4 mm), sagittal intermediate-weighted turbo-spin echo sequence (TR/TE 2890 ms/33 ms; FOV 160 mm; matrix 256 × 256; slice thickness 2 mm), transverse short tau-inversion recovery sequence (TR/TE 5000 ms/62 ms; inversion time 210 ms; FOV 180 mm; matrix 320 × 320; slice thickness 7 mm), transverse T1-weighted turbo-spin echo sequence (TR/TE 820 ms/13 ms; FOV 180 mm; matrix 448 × 448; slice thickness 6 mm). Additionally, in all postoperative patients a transverse Dixon sequence was acquired (TR/TE 3.9 ms/1.2 ms; FOV 200 mm; matrix 128 × 128; slice thickness 5 mm) and based on this Dixon sequence fat fraction maps were calculated on the scanner for intramuscular fat quantification.

### Statistical analysis

Statistical analysis was performed using Mann-Whitney U-test when investigating HHS, VAS, Western Ontario and McMaster Universities Osteoarthritis Index (WOMAC), hip abductor strength and Fischer Exact Test when comparing the need for daily painkiller consumption. All statistical tests were 2-tailed. The alpha level was set at 0.05. Standard statistical methods were used for descriptive statistics Data were expressed as mean ± standard deviation (SD) for quantitative variables and as percentages for qualitative variables.

## Results

A total of 8 patients met the inclusion criteria. No patient was lost to follow-up. Mean follow-up was 35 months (range 26–54 months; SD 9.7). Of the 8 patients, 6 were female, the average age at surgery was 69 years (SD 4.7) and average body mass index (BMI) was 26 kg/m^2^ (SD 3.4); three patients were active smokers with an average of 52 pack years (range 15–100). The left hip was involved 4 times.

### Clinical results

Clinical findings preoperatively and postoperatively are summarised in Tables 1 and 2, respectively.

**Table 1.** Patient demographics and preoperative clinical findings.

#	Age [years]	BMI [kg/m^2^]	THA	Side	Trendelenburg gait	Trendelenburg sign	Hip abductor strength (0–5)^a^	Cane/ Crutches	Pain level (VAS)	Pain killers daily
1	67	31	Yes	R	Yes	Yes	2	Cane	5.5	No
2	68	24	No	L	Yes	Yes	2	Crutches	4.5	Yes
3	70	23	Yes	R	Yes	Yes	2	No	5.5	Yes
4	75	28	Yes	R	Yes	Yes	3	No	5	No
5	75	25	No	R	Yes	Yes	3	No	2	No
6	63	20	Yes	L	Yes	Yes	2	No	7	Yes
7	63	29	Yes	L	Yes	Yes	2	Cane	7.5	Yes
8	74	23	Yes	L	Yes	Yes	3	Cane	5.5	yes

BMI, body mass index; THA, total hip arthroplasty; VAS, visual analogue scale (0 = no pain, 10 = maximal pain).

a MRC, Medical Research Council score.

**Table 2.** Clinical findings postoperative.

No.	Follow-up (months)	Do surgery again?	Trendelenburg gait	Trendelenburg Sign	Hip abductor (strength grade)^a^	Cane/ Crutches	Pain level (VAS)**	Pain killers daily
1	54	yes	Yes	Yes	2	Cane	4.5	No
2	47	yes	No	Yes	4	No	0	No
3	38	no	No	Yes	2	Cane	2	No
4	33	yes	Yes	Yes	4	Cane	0.5	No
5	30	no	No	Yes	4	Cane	4.5	No
6	27	no	Yes	Yes	1	No	6.5	No
7	27	yes	Yes	Yes	4	Cane	5.5	No
8	26	yes	No	Yes	4	Cane	0	No

VAS, Visual analogue scale (0 = no pain, 10 = maximal pain).

a MRC, Medical Research Council score.

Postoperatively the patients reported significantly less pain (VAS 2.5 [SD 2.4] vs. 5 [SD 1.6], *p* *=* 0.046) and none of the patients needed pain killers on a daily basis compared to 5 patients preoperatively (*p* *<* 0.026). However, 1 patient (#5) reported an increased pain level from VAS 2 to VAS 4.5, albeit in this patient hip abductor strength increased from M3 to M4. 3 of the 8 patients would not have the surgery again (# 3,5,6).

Trendelenburg gait was present in 7 of 8 patients preoperatively and only in 4 of 8 patients postoperatively. However, Trendelenburg sign remained positive in all patients postoperatively. Hip abductor strength did not increase significantly from an average of 2.4 (SD 0.48) to 3.1 (SD 1.17; *p* *=* 0.19). The greatest strength increase was 2 points from M2 to M4 (#2). 1 patient had a decrease in strength postoperatively from M2 to M1 (#6), this is the same patient who required a surgical debridement including antibiotic therapy due to a wound healing disorder. No other complications were noted. HHS and WOMAC score did not differ significantly postoperatively, HHS increased from 42 (SD 3.63) to 63.3 (SD 20.2) and WOMAC decreased from 5 (SD1.55) to 3.81 (2.02).

### Radiographic results

Pre- and postoperative MRI findings are summarised in Table 3. Intraoperative findings were consistent with preoperative MRI diagnosis in all 8 patients. Trochanteric bursitis was evident in 7 of 8 patients preoperatively. No trochanteric bursitis was seen postoperatively.

**Table 3.** Radiographic results pre- and postoperative (MRI).

No.	Integrity of gluteal tendons	Trochanteric bursitis/ oedema^a^	Muscle atrophy preoperative^b^			Fatty degeneration preoperative^c^		
			Glut. Max.	Glut. Med	Glut. minim	Glut. Max.	Glut. Med	Glut. minim
1	complete avulsion	2	0/0/0	1/1/1	1/2/2	2/2/2	2/3/3	3/2/2
2	complete avulsion	1	0/0/0	0/1/1	1/1/1	1/1/1	1/3/1	1/3/1
3	complete avulsion	2	0/0/0	1/2/1	2/2/2	2/1/1	3/4/1	3/4/3
4	complete avulsion	2	0/0/0	1/2/1	2/3/2	2/2/2	1/3/2	2/3/2
5	complete avulsion	2	0/0/0	1/2/2	2/3/2	2/2/2	2/4/3	2/4/2
6	complete avulsion	2	0/0/0	1/1/1	2/2/2	2/2/2	1/3/2	2/2/2
7	complete avulsion	-	0/0/0	2/1/1	2/3/2	2/2/2	3/3/2	2/4/3
8	complete avulsion	2	0/0/0	2/2/1	3/3/2	1/1/1	3/3/1	3/4/1
No.	Integrity of the tendon transfer	Follow- up [months]	Muscle atrophy postoperative			Fatty degeneration postoperative		
			Glut. Max.	Glut. Med	Glut. minim	Glut. Max.	Glut. Med	Glut. minim
1	Yes	**54**	0/0/0	1/1/1	1/2/2	2/2/2	2/4/4	4/4/4
2	Yes	**47**	0/0/0	0/1/1	1/1/1	1/1/1	1/3/1	1/3/1
3	Yes	**38**	0/0/0	2/2/1	2/2/2	2/1/1	4/4/2	3/4/3
4	Yes	**33**	0/0/0	1/2/1	2/3/2	2/2/2	2/3/2	2/3/2
5	Yes	**30**	0/0/0	1/2/2	2/3/2	2/2/2	2/4/3	2/4/2
6	Yes	**27**	0/0/0	1/1/1	2/2/2	2/2/2	1/3/2	2/2/2
7	Yes	**27**	0/0/0	2/2/1	2/3/2	2/2/2	3/4/2	2/4/3
8	yes	**26**	0/0/0	2/2/1	3/3/2	1/1/1	3/4/2	4/4/2

a 0 = normal, 1 = oedema, 2 = circumscribed fluid collection.

b 0 = normal, 1 = mild atrophy 2 = moderate atrophy 3 = severe atrophy.

c Modified Goutallier classification.

After a mean follow-up of 35 months (26–54 months), complete healing of the tendon transfers was confirmed by MRI. There were no re-ruptures.

Comparing the MRI findings pre- and postoperatively, there was no deterioration of the gluteus maximus volume or increased fatty degeneration in both the retained posterior and the transferred anterior part. Increased atrophy of the gluteus medius was seen in 2 patients, and an increased fatty degeneration in 5 patients. An increased fatty degeneration in the gluteus minimus was seen in 2 patients.

## Discussion

The most important finding of this study is that by reinforcing the hip abductor reconstruction with an anterior M. gluteus maximus flap,^14^ a salvage procedure in the treatment of painful chronic hip abductor insufficiency, a significant pain reduction from a mean VAS of 5 preoperatively to 2.5 postoperatively (*p* *=* 0.046) was achieved with a reduction in all but one patient after a minimum follow-up of 26 months (mean 35 months). Additionally, none of the patients had to take painkillers on a daily basis postoperatively compared to 5 patients who required pain killers daily preoperatively. Although HHS and WOMAC improved non-significantly, these changes may reflect a subjective benefit, but still with limited hip function.

An effect of the muscle transfer on strength was measurable but modest and not significant (M 2.5 (SD 0.7) to M 3.13 (SD 1.2)). We attribute this effect mainly to the pain relief and consequently possibly increased activation of the reattached hip abductors rather than to an actual favorable force development of the gluteus maximus muscle transfer. In our opinion, the force vector of the gluteus maximus transfer is not optimal to replace the function of the hip abductors, however, we agree that there are no reasonable alternatives. Clinically the improvement in hip abductor strength was negligible as none of the patients were able to sufficiently stabilise their pelvis in a 1-legged stance resulting in 100% positive Trendelenburg sign pre- and postoperatively. Only 1 patient was able to ambulate longer distances without a cane or crutches postoperatively (# 2). These results are comparable to the 6 patients described by Whiteside and Roy^22^ with a similar detrimental situation preoperatively and comparable to a previous study performed at our institution investigating the advancement of the vastus lateralis muscle for chronic hip abductor insufficiency.^29^

3 of the 8 patients would retrospectively not have the procedure done again. 1 patient had an increased pain level 30 months after the index surgery, 1 patient had a minimal pain relief of 0.5 points on the VAS score and remained dependent on a walking aid and the third patient had a marked decrease of the pain level from VAS 4.5 to 2 but remained severely limited with an unvaried hip abductor strength of M2. It is possible, that these patients had a higher expectation in the performed surgery. We believe it is thus critical to emphasise that the goal of the surgery is a pain reduction and not an improvement of hip abductor function.

**Figure 2.**
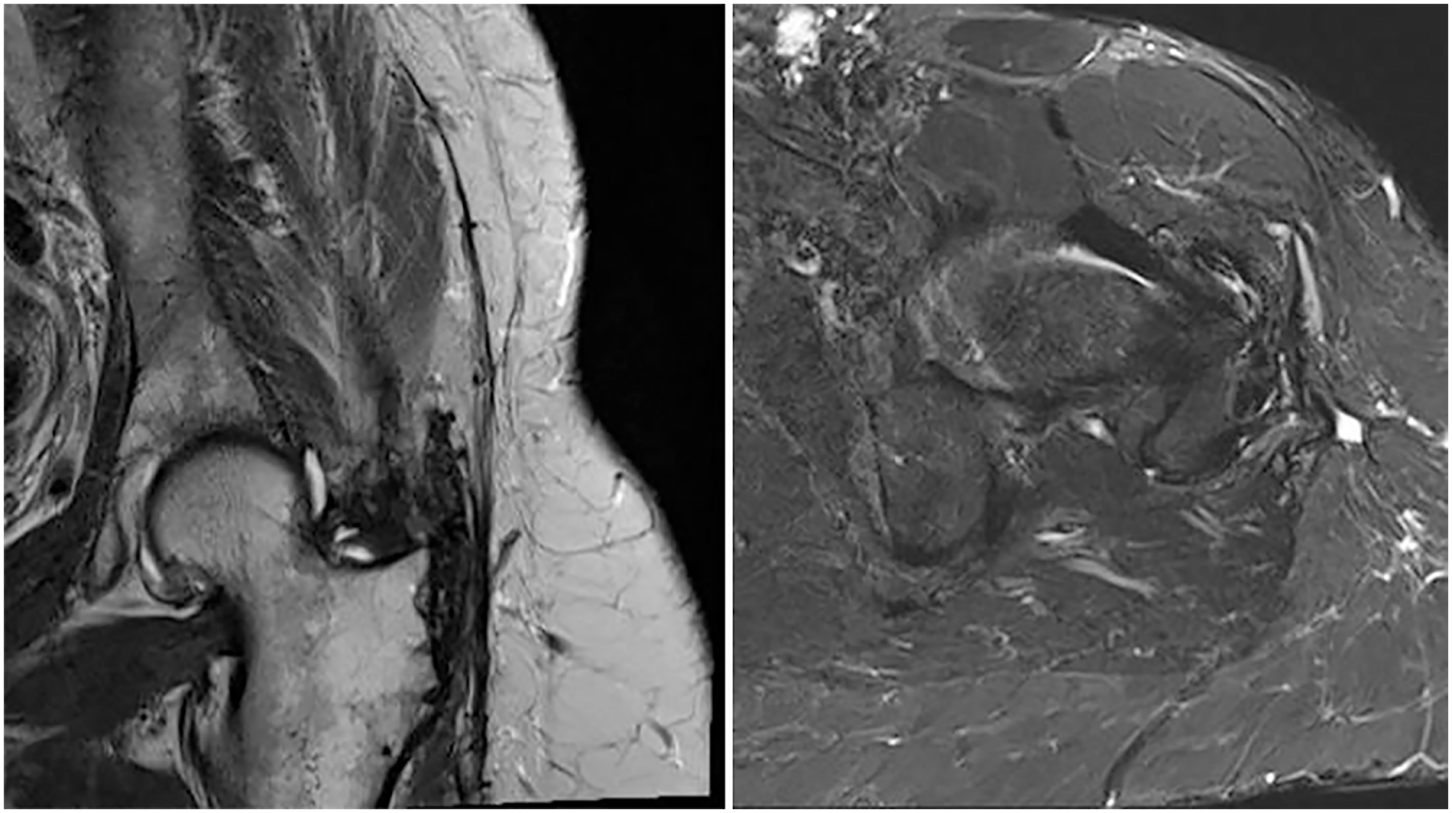
MR image depicting structural integrity of the extraanatomical tendon repair at a follow-up more than 2 years postoperatively.

To our knowledge this is the first study including pre- and postoperative MRI assessment after gluteus maximus tendon transfer. We can thus confirm that the extraanatomical tendon transfer showed structural integrity in all patients after a mean follow-up of 35 months (26–54 months). No re-ruptures were noted. No trochanteric bursitis was seen postoperatively (Figure 2). The fatty degeneration of the native hip abductors, however, was not stopped. The progressing degeneration may show the natural course or may reflect a possible additional iatrogenic damage due to the surgery. The transferred as well as the retained gluteus maximus muscle showed no change regarding atrophy or fatty degeneration, confirming the mid-term survival of the segmentally innervated and perfused muscle. The higher proportion of fatty streaks in the healthy gluteus maximus muscle compared to the gluteus medius muscle is a known phenomenon.^30,31^

Our patient outcomes are overall inferior to the results published by Christofilopoulos et al.^20^ While the salvage surgeries achieved a significant pain reduction in both studies, the patient group of Christofilopoulos started with a considerably greater pain level of VAS 7–8 preoperatively potentially due to a marked inflammation of the abductor tendons. Our results are markedly inferior regarding a lesser increase of hip abductor strength from M2.4 to M3.1, while Christofilopoulos reports a significant increase from M3 to M4, starting with greater preoperative strength.^20^ A strength grade of M2 is markedly inferior to M3.

Our results are inferior regarding postoperative Trendelenburg sign which persisted in all of our patients compared to only 12/38 patients (32%) with persistent Trendelenburg sign in their study, as well as regarding Harris Hip Score which is markedly lower in our study (63 points) compared to 80 points.

There may be several explanations for this difference. The most likely explanation is that the pathology of our patient group differed from their group, as all of our patients had a complete avulsion of the hip abductor tendons as confirmed by MRI. The conservative treatment regimen is not specified in their study and might have not included steroid infiltration to the trochanteric bursa influencing the preoperative assessment. In their study it is not specified if a complete avulsion of the hip abductor tendons was present intraoperatively and thus a reflective painful weakness of the hip abductors as seen with marked inflammation may have been the reason for the high pain level preoperatively. As a consequence of the marked pain reduction, the patients were able to develop more strength by activating more muscle fibers of the hip abductors without the transfer clearly contributing to the strength. As a consequence most of the patients showed a negative Trendelenburg sign after reduction of the pain. It is thus possible that some of these patients could have been treated satisfactorily with a steroid infiltration.^10^ Additionally, our mean follow-up of 35 months is considerably longer than the mean follow-up of 19.2 months and deterioration of the function due to further deconditioning or aging is possible.

Chandrasekaran et al.^21^ reports similar results to our study in a small cohort of 3 patients. They summarise that the transfer of the gluteus maximus leads to a relevant decrease in pain but a minimal effect on hip abductor strength.

In summary, we will continue to perform gluteus maximus transfers as a salvage procedure in this rare and difficult clinical situation as we are not aware of more favourable alternatives. It is of great importance to realistically manage patient expectations preoperatively. A significant pain relief can be achieved in most patients, while the effect on hip abductor function and gait remains very limited.

Limitations of this study include its retrospective design, lack of control group and small sample size for this rare condition. However up to date, it remains the largest consecutive series of patients with a complete pre- and postoperative MRI evaluation and a minimal follow-up of 24 months.

## Conclusion

In the setting of painful chronic hip abductor insufficiency refractory to conservative treatment with advanced muscle degeneration without the possibility of a direct reconstruction the gluteus maximus tendon transfer significantly decreased pain. The effect on hip abductor strength and patient reported functional outcome scores is limited. Despite the modest results it remains our preferred salvage treatment option for lack of better alternatives. Larger studies are necessary to confirm these findings.
